# Local and non-local effects (on the posterior chain) of four weeks of foot exercises: a randomized controlled trial

**DOI:** 10.1038/s41598-024-71585-y

**Published:** 2024-09-23

**Authors:** Anna Gabriel, Sarah T. Ridge, Michael Birth, Thomas Horstmann, Torsten Pohl, Andreas Konrad

**Affiliations:** 1 Professorship for Conservative and Rehabilitative Orthopedics, School of Medicine and Health, Technical University of Munich, Munich, Germany; 2 Department of Rehabilitation Sciences, College of Education, Nursing and Health Professions, University of Hartford, West Hartford, CT USA; 3 Institute of Human Movement Science, Sport and Health, Graz University, Graz, Austria

**Keywords:** Superficial backline, Force transmission, Intrinsic foot muscles, Dorsal chain, Plantar foot sole, Orthopaedics, Randomized controlled trials

## Abstract

This study investigated the local, remote, and contralateral effects of a four-week intrinsic foot muscle exercise intervention in recreationally active participants on foot parameters, flexibility, and performance of the posterior chain (PC). Twenty-eight healthy participants (12f, 16m) were randomly assigned to a control group or performed 2 × 6 min of foot exercises twice daily unilaterally at least five days/week for four weeks. At baseline (M1), after the intervention (M2), and after a four-week wash-out period (M3), we assessed bilateral Foot Posture Index-6, medial longitudinal arch mobility, single-leg stance balance, range of motion (ROM) (first metatarsophalangeal joint and ankle), and flexibility and performance of the PC. The FPI-6 score changes over time differed significantly between groups for both legs, improving by 26% in the trained- (p < .001) and 11% in the untrained leg (p = .02) in the intervention group from M1 to M2. Improvements were maintained at M3 for the trained leg (p = .02). Ankle range of motion and balance of the trained leg improved from M1 to M2, yet only became significant at M3 (ROM: p = .02; balance: p = .007). The other parameters did not change significantly. A four-week foot exercise intervention might have local but no remote effects in healthy young adults.

**German Clinical Trial Register** (DRKS00027923) (24/08/2022).

## Introduction

The intrinsic foot muscles (IFM) play a crucial role in supporting and maintaining the stability of the medial longitudinal arch during movement^1,2^. The plantar IFM are organized into four layers, with the most superficial layer being functionally connected to the plantar fascia^3,4^. Dysfunction or weakness of the IFM are associated with an increased risk for foot dysfunctions, like plantar fasciitis, foot deformities, a reduced medial longitudinal arch support or flexibility, and an increased risk of injury^5–10^.

For the conservative therapy of foot disorders, or prevention of such, a training program of IFM foot exercises (FE) is recommended^11^, which commonly consists of the toe spread exercise, short-foot exercise, isolated extension of the hallux, and the lesser toes, and the toe-towel curl^4,12^. Studies report that FE interventions, ranging from four to 16 weeks, improve medial longitudinal arch mobility, foot posture, balance ability, motor activation, and motor performance, increase toe flexion strength and muscle cross-sectional area, and change foot biomechanics during running in healthy participants and patients with flatfoot^1,3,4,12–21^.

The foot structures and posture have also been discussed to influence remote body areas of the lower extremity and trunk^22,23^. There is evidence for remote effects on flexibility along the posterior chain (PC) after foam rolling, stretching, or manual technique interventions on the plantar foot sole. The PC was described as comprising the myofascial structures from the plantar foot sole over the dorsal leg to the lower back in accordance with the superficial backline, which—in contrast—continues over the upper back and neck to the posterior head structures^24–27^. Transmission effects along myofascial connection or neurophysiological effects are discussed as a potential mechanism for the findings^28–31^. For active interventions on the plantar foot sole (e.g. minimalist shoe walking or FE), some studies also report remote effects along the PC, which might be due to biomechanical factors (e.g. different walking/running pattern), while other studies do not support these findings^32–38^. Sulowska, et al.^38^ and Sulowska-Daszyk, et al.^36^, for example, showed that short foot exercises performed for six weeks also could improve remote flexibility and strength along the PC in runners. It should be noted that in their studies^36,38^, runners progressed the FE from sitting to standing and performed other strengthening exercises (e.g., Forward lean) in a standing position. Further, participants only did the short foot exercise (not a total bout of FE, including e.g., toe spread exercise), which might also have influenced the results^36–38^. To our knowledge, no studies have investigated the chronic remote effects of a total bout of IFM FE, which only performed in a sitting position and without additional exercises, on the PC in a recreationally active population. Further, there is no evidence on contralateral chronic effects of FE.

Therefore, this study investigates the effect of a four-week FE intervention on foot posture, medial longitudinal arch rigidity, local range of motion (ROM), remote PC flexibility, and PC performance in healthy, recreationally active participants. Furthermore, we investigate potential exercise crossover effects by conducting the intervention unilaterally. In addition, we report observed effects after a four-week wash-out period. We hypothesize that four weeks of FE, impact foot parameters (i.e., posture and medial longitudinal arch mobility), local ROM (i.e., toe and ankle) and remote PC flexibility and performance (i.e., isometric and concentric). We think it is unlikely that these effects can also be seen on the contralateral leg and remain after a four-week wash-out period.

## Materials and methods

This two-arm parallel-group blinded randomized controlled trial was conducted in accordance with the ethical principles of the Declaration of Helsinki as part of a larger randomized controlled trial, which is registered in the German Clinical Trial Register (DRKS00027923) and approved by the ethics committee of the Technical University of Munich (2022-114-S-KK). Participants provided written informed consent before the study. The study followed the CONSORT guideline for randomized controlled trials.

### Participants

We included 30 healthy participants (14 female, 16 male), who were classified as at least low to moderately active, i.e., walking a weekly average of at least 5000–10,000 steps a day^39^ (tracked by participants via smartphone app (Accupedo, Corusen, US)).

Participants were not allowed to participate in this study if they were regularly wearing minimalist shoes (MS), were professional athletes, were pregnant, or were in the nursing period. We excluded persons with diagnosed (childhood) foot deformities, pain in the elbow, shoulder, back, leg, or foot area, musculoskeletal injury in the lower extremity or the lower back in the last 12 months, a history of surgery in the lower limb or lower back, self-reported impairment that would affect general motor function, balance, blood circulation, sensitivity or pain sensation.

We estimated the sample size with G*Power (G*Power version 3.1., Heinrich-Heine-University Düsseldorf, Germany) based on the outcome foot posture (FPI-6) and the reported effect sizes (ES) of prior studies on FE interventions for six weeks in runners (standing FE)^37^ and in patients with pes planus (only short foot exercise)^14^. We assumed a within-group standard deviation (*sd*) of 1.5 points and a relevant difference between the groups of 2 points^14^, translating to an effect size of Cohen’s *d* = 1.3, converted to Cohen’s *f* = 0.7^40^ (for ANOVA). For assumed *α* = 0.05, and *β* = 0.95, the estimated sample was n = 29, resulting in *n* = 15 per group. Our sample size appears to be similar to prior FE studies^12,37^.

### Study procedure

We recruited participants in the university setting. After checking the inclusion criteria, the study lead randomly assigned participants to the control and FE group via online random sequence generator^41^. The examiners were blinded concerning the study group allocation and participants were not told about the other group's intervention during the study.

We took measurements at baseline (M1), after the four-week intervention (M2), and after the four-week wash-out period (M3), which all took place in the laboratory of the Technical University of Munich (Germany). Before each measurement, we instructed participants to avoid strenuous physical activity and excessive consumption of alcohol or stimulants in the 24 h before the test sessions. We provided them with video material to familiarize them with the measurement methods. At M1, the study lead randomly defined either the dominant leg (preferred leg for kicking a ball)^42^ or the non-dominant leg as the first leg to be measured via an online random sequence generator^41^. After a five-minute warm-up with an ergometer at 80W at a self-selected speed, all participants went through the testing battery in the same order. After M1, participants of the FE group received verbal and printed instructions on the intervention. During the intervention period, they had access to video instructions via a web-based training software (Lanista, MP Sports, Coaching & Consulting GmbH, Germany), with which they also documented the training process. All measurement sessions were taken at the same time of day and lasted approximately one hour.

### Active foot strengthening exercises

**Fig. 1:**
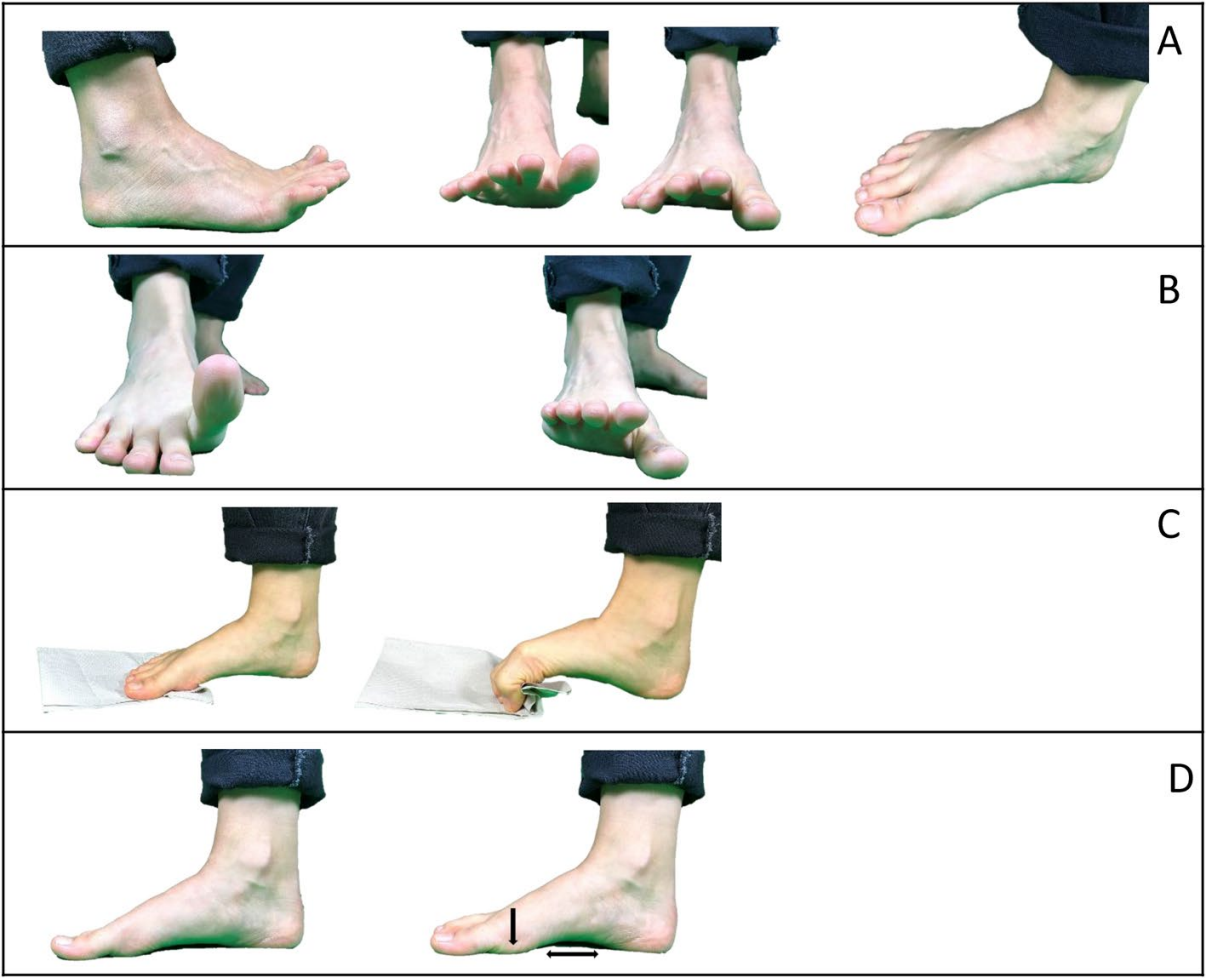
The foot exercise intervention: Starting with (**A**) a warm-up sequence, followed by either (**B**) hallux- and lesser toe extension or (**C**) the short-foot exercise and (**D**) toe grip exercise.

While the control group performed no intervention, participants of the FE group were instructed to perform five FE (Fig. 1), which mainly targeted the IFM and were investigated in prior studies^1,3,4,12,14,15,17–19,38^. Participants were asked to perform the FE with a randomly chosen leg (by study lead via online sequence generator^41^) while barefoot in a sitting position. The exercises were split into two bouts (around 6 min each), performed at different times of day (i.e., Block 1: morning or pre-noon, Block 2: afternoon or evening) and at least five days per week. Each session started with a warm-up (Fig. 1A), the toe-spread-out exercise. After that, participants performed the respective Block of FE according to daytime: Block 1 consisting of the hallux- and lesser toe extension exercises (Fig. 1B); Block 2 consisting of the toe curl (Fig. 1C) and the short foot exercise (Fig. 1D). All exercises were repeated ten times. In exercises B, C, and D, the final position was held for 5 s and repeated for three sets with a 10-s pause.

After one week, we contacted the participants via telephone to answer any potential uncertainties regarding the intervention and improve training compliance. If participants managed to perform FE and there were no adverse events or negative side effects (i.e., pain), they were asked to increase the training intensity by applying resistance with an elastic band (TheraBand, Ludwig Artzt GmbH, GE). The resistance band (intensity: red) was either put over or under (i.e., extension or flexion) the big toe or the lesser toes, and participants held the band ends in both hands to apply resistance to a maximum so that they were still able to perform the exercise to a full range of motion and the pre-given repetitions. The band was placed under the metatarsophalangeal joint for the short foot exercise. Participants applied tension to the band (with one hand) and conducted the training with the aim of being unable to pull out the band.

### Measured outcomes

All outcome parameters were measured on both legs. The quantitative outcome variables were the Foot Posture Index-6 (FPI-6), the Arch Rigidity Index (ARI), balance ability during single-leg stance, ROM of the first metatarsophalangeal joint and the ankle, as well as flexibility and performance of the PC, namely the Bunkie Test, the Standing 90:20 Isometric Posterior Chain Test and the isokinetic, concentric measurement of the hamstrings.

#### Foot parameters

##### Foot posture index 6

The FPI-6^43^ includes six items to evaluate the forefoot and the rearfoot components in the three cardinal body planes. The scoring system uses a five-point Likert-scale where lower scores represent a more supinated foot and higher scores represent a more pronated position. For further analysis, we changed the original score (− 2 to 2) to the adapted score (1 to 5)^44^, where 3 indicates a neutral foot position. Participants were instructed to jump three times and then stand relaxed for the scoring. The same experienced examiner performed all FPI-6 measurements.

##### Arch rigidity index

The ARI, which measures the structural mobility of the medial longitudinal arch^3^, is calculated as the standing Arch Height Index divided by the sitting Arch Height Index. The Arch Height Index itself is determined by dividing the height of the dorsum of the foot by the truncated length of the foot^11^. The ARI is typically measured using a specialized system as described by Mulligan and Cook^3^ and Tourillon, et al.^11^. For the sitting Arch Height Index, participants sat with hips and knees at 90° flexion and the subtalar joint in neutral position. The foot was placed on a device, on which the total (calcaneus to tip of longest toe) and truncated (calcaneus to centre of the first metatarsal head) foot length were measured with a fixed cloth tape. The dorsum foot height was measured with a modified carpenter’s square at the 50% mark of the foot length. For the standing Arch Height Index, participants stood on one leg, holding on to a wall for support, and the examiners checked and corrected for any hip abduction.

##### Balance during single-leg stance

Static balance, as an indirect measurement of IFM function, was quantified by evaluating the center of pressure (CoP) during a single-leg stance. Participants were instructed to stand as still as possible for 30 s on a pressure platform (RSScan, Logemas, AUS), looking straight ahead with the other knee flexed at 60°. Their hands rested on their hips. Data were only recorded in the final 25 s (sampling frequency: 500 Hz), with a 5-s delay at the outset to control for any initial balance irritations. Then, after a 30-s pause, in which participants could walk a few steps, the second leg was tested^1,15^. The path length in mm and the ellipse area of the CoP in mm^2^ (as calculated by the software) were included in the statistical analysis. The elliptical area includes all coordinates that are within one standard deviation in the x and y directions around the origin.

#### Range of motion and flexibility

##### First metatarsophalangeal joint

To evaluate the MTPJ1 ROM, passive maximum dorsiflexion was quantified using a clinical goniometer (Model 01135, Lafayette Instruments Co., Sagamore, IN, USA). Participants were instructed to perform a reverse lunge with the tested leg moving backward while maintaining hand contact with a wall to provide stability. In the lunge position, the participants were then directed to lift their heel of the rear leg while keeping the MTPJ1 in contact with the floor to induce a passive dorsiflexion^45^. The angle between the shaft of the first metatarsal bone (movable arm) and the floor (fixed arm) was then measured to the nearest degree^45,46^. The maximum value of three consecutive trials was recorded.

##### Ankle joint

Ankle ROM was assessed with the knee-to-wall test^47^. Participants were instructed to take a lunge position with the test leg in front, positioning the big toe 10 cm away from a wall. A standard tape measure, fixed on the floor perpendicular to the wall, ensured accurate distance. Participants were then instructed to touch the wall with the knee of the tested leg while keeping the heel in contact with the floor. The tape measure also controlled for heel alignment, and an extension of the tape at the wall was used to ensure proper knee alignment, with the participants being instructed to move their patellar to the tape. To further standardize the test and to control for heel lifting, a stretched elastic band (TheraBand, Ludwig Artzt GmbH, GE) was placed under the participants' heels with one end fixed on the ground. If participants could touch the wall with the knee, they progressively placed their foot further away one cm at a time and repeated the lunge until they could not touch the wall with their knee without lifting the heel off the ground. Ankle ROM was quantified as the distance of the big toe from the wall in cm.

##### Posterior chain

The modified back-saver sit-and-reach test is an alternative to the classical sit-and-reach test, which is commonly applied to assess the flexibility of the hamstring and lower back. We chose this version of the sit-and-reach test as it allows to test one leg at a time, considers leg length discrepancies, and prevents discomfort in the contralateral hip joint^48^. For the test, participants sat on a bench of approximately 30 cm in height and placed the foot of the tested leg on the standardized sit-and-reach box. The other leg was placed on the floor with knee flexed approximately 90°. They performed three trials per leg, where the best score was used for further analysis.

#### Performance posterior chain

##### Standardized Bunkie Test

Participants were instructed to place their forearms on a mat in supine position with the shoulders over the elbows and the heels placed on a box (30 cm), with both legs straightened^24,49^. They performed a reverse plank by lifting the pelvis to a neutral position, and then raised the contralateral leg about 10 cm off the box to assess one leg. To standardize the testing procedure, the horizontal pelvis position was marked with a rubber band stretched between two fixed bars and the contralateral lifted foot height was marked by a box (height 10 cm, placed directly in front of the feet) according to Gabriel, et al.^24^ or Gabriel, et al.^25^. The time of the correctly maintained position was counted with a stopwatch (in s) and indicated the test performance. If participants reported burning, cramping, pain, or strain, or reached the cut-off score of 40 s^49^, this ended the test. Further, they could end the test due to fatigue. If participants could not maintain the correct test position, the examiner verbally corrected them. They were allowed to correct the position once per body area. Further deviations beyond the first correction also ended the test. After a 30-s pause, the test was repeated for the other leg^49–52^. The same experienced examiner performed all Bunkie Test measurements.

##### Standing 90:20 isometric posterior chain test

Participants stood with their legs and lower back against a wall and crossed their arms in front of their chest with the hands placed on the shoulders. They were instructed to place one leg (tested leg) with neutral ankle flexion on a force plate (FP4060-10-TM-2000, Bertec, Columbus, Ohio, USA). The force plate rested on a standing desk with adjustable height, which was adjusted so participants were tested in 90° hip flexion and 20° knee flexion position. Before the measurement, joint angle positions were checked with a goniometer and participants were instructed to keep the standing leg extended and in contact with the wall, which was controlled for by the examiner^53^.

The examiner instructed participants ‘to exert maximal force into the force plate (i.e., to the ground/table)’, and participants were verbally encouraged for 5 s. The right and left legs were tested alternatingly (3 times each) with a 60-s pause in between. The force plate sampling frequency was set to 1000 Hz. Torque values (Nm) were captured with proEMG (prophysics AG, CH) and smoothed with a 20-ms moving average. The average of each limb's vertical maximum torque values was used for further analysis.

##### Isokinetic measurement of the hamstrings

The knee flexors muscle strength during concentric movement (ROM 5°–90°) was evaluated via isokinetic testing (ISOMED 2000, D & R Ferstl GmbH, CH). Participants were informed about the measurement procedure in advance. Then, they sat on the device and were fixed with straps over the shoulders, across the waist, and over the middle of the thigh, and the dynamometer arm's rotation axis was positioned lateral to the femoral epicondyle. Before each trial, participants completed a test trial with five repetitions at sub-maximal force to familiarize themselves with the movement and speed. The test was performed for five repetitions at 60°/s followed by five at 120°/s with a one-minute pause between, and then repeated for the other leg^54–57^. The angle and torque values (Nm) were captured with proEMG (prophysics AG, CH) at 1000 Hz and processed in Matlab (R2020b, MathWorks, US). For the final analysis, we calculated the average of the maximum flexion peak torque from repetitions two to four (out of five) for both angular speeds.

### Statistical analysis

Statistical analysis was performed using the statistical software R (version 3.5.1, R Core Team, AUT)^58^. Each outcome parameter was calculated separately for trained and non-trained leg. Participants' characteristics and descriptive data of the outcome parameters are presented as mean ± sd. To analyze the respective outcome parameters, we checked the required assumptions and excluded potential outliers. We calculated a linear regression model using the lmer-package^59^ with the fixed variables group (intervention, control) and measurement (M1, M2, M3) and the random variable participant. Further, we performed a second linear regression analysis evaluating the interaction between group and measurement and compared the two models with a likelihood-ratio test using analysis of variance (ANOVA). We performed the analysis for the trained leg and a randomly allocated leg of the control group and present the same analysis for the untrained leg separately. A p-value of ≤ 0.05 was considered significant.

## Results

### Participants

**Fig. 2:**
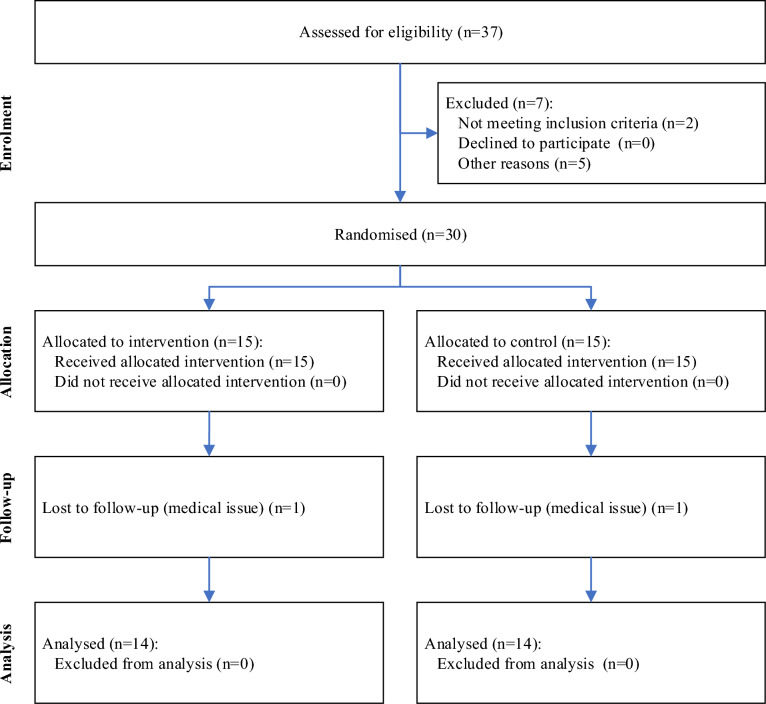
CONSORT study flow diagram.

After a drop-out of two female participants (1 control group, 1 FE group) due to health reasons, our final sample consisted of 28 participants. The CONSORT study flow diagram is presented in Fig. 2. All participant characteristics variables were normally distributed (all p ≥ 0.05) and are listed in Table 1. Participants of the FE group performed the intervention on (mean ± sd) 5.3 ± 1.4 days per week. There were no self-reported adverse or unwanted side effects (i.e., pain) due to the intervention.

**Table 1:** Participants’ characteristics at baseline.

Variable (mean ± sd)	Total n = 28	Active group n = 14	Control n = 14
Age	25.90 ± 4.34	27.20 ± 3.49	24.50 ± 4.80
Weight	71.90 ± 13.80	73.50 ± 14.10	70.20 ± 13.80
Height	176.00 ± 8.74	177.00 ± 9.55	175.00 ± 8.17
Steps/day	8395 ± 2971	7613 ± 3238	9177 ± 2555
(n =)			
Sex	12f./16 m	4f./10 m	8f./6 m
Dominant leg	8le/20ri	3le/11ri	5le/9ri
Trained/first measured leg	do:4le/14ri n-do:7le/3ri	do:2le/9ri n-do:2le/1ri	do:2le/5ri n-do:5le/2ri

*sd* standard deviation, *m* male, *f* female, *le* left, *ri* right, *do* dominant leg, *n-do* non-dominant leg.

### Foot parameter

**Fig. 3:**
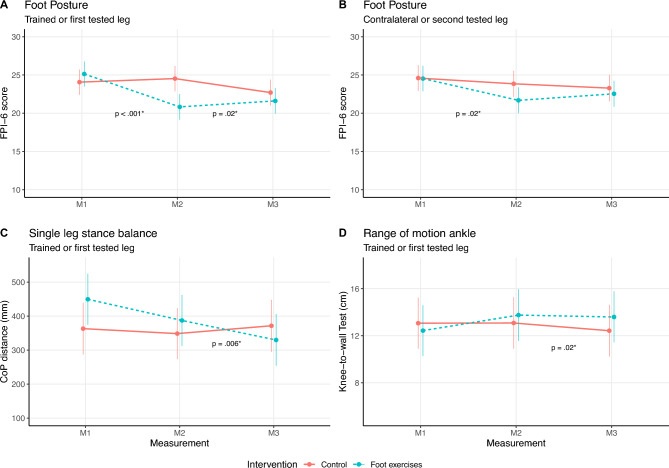
The effect plots of the linear regression results for the results of (**A**) the Foot Posture Index-6 for the trained and (**B**) the contralateral leg, (**C**) the single leg stance balance, and (**D**) the range of motion of the ankle. Displayed p-values denote significant differences between groups in the changes between time points. *Note* the y-axis does not start at 0.

Concerning the FPI-6, for the trained leg, the likelihood-ratio test indicated that the model including the interaction between group and measurement time point provided a better fit for the data than a model without it (χ^2^(2) = 24.40, *p* < .001), similar to the untrained leg (χ^2^(2) = 6.02, *p* = .05), which means that the FPI-6 score changes between the measurement points are significantly different between groups. For the trained leg, there was a significantly higher decrease of 26% in the FPI-6 score between M1 and M2 for the FE group (β = − 4.77 (standard error (SE) = 0.9)) compared to the control group, where the FPI-6 score stayed the same (24.0 ± 3.8) (t(52) = − 5.27, p < .001)) (Table 2). Also, the change over time from M1 to M3 differed significantly between groups (t(52) = − 2.35, p = .02) with a reduction of around 24% for the FE group (Table 2). For the untrained leg, there was also a decrease from M1 to M2 of about 11%, which differed significantly from the control group (β = − 2.10 (0.88)) (t(52) = − 2.38, p = .02), but not from M1 to M3 (Table 2). The effect plots of the FPI-6 linear regression results are shown in Fig. 3.

**Table 2:** Descriptive data of the foot parameters for both groups (foot exercise and control) at all three measurement points.

Variable	Measurement	Foot exercise (mean ± SD)		Control (mean ± SD)	
		Trained	Untrained	First tested	Second tested
FPI-6 score	M1	25.1 ± 2.4	24.5 ± 2.9	24.1 ± 3.8	24.6 ± 3.5
	M2	20.8 ± 3.3	21.7 ± 3.1	24.8 ± 3.0	24.1 ± 2.9
	M3	21.6 ± 2.8	22.6 ± 3.2	23.0 ± 3.3	23.6 ± 3.4
ARI score	M1	0.92 ± 0.05	0.93 ± 0.03	0.92 ± 0.04	0.92 ± 0.04
	M2	0.92 ± 0.05	0.94 ± 0.04	0.94 ± 0.04	0.94 ± 0.05
	M3	0.93 ± 0.05	0.93 ± 0.04	0.92 ± 0.04	0.92 ± 0.04
CoP path length (mm)	M1	449.6 ± 170.8	409.1 ± 196.3	359.4 ± 105.5	348.4 ± 109.2
	M2	387.2 ± 132.6	413.8 ± 254.6	348.6 ± 139.1	337.5 ± 124.8
	M3	335.8 ± 118.7	363.7 ± 157.9	377.2 ± 161.2	339.5 ± 95.0
CoP EA (mm^2^)	M1	39.6 ± 16.3	47.3 ± 29.1	33.7 ± 12.8	30.4 ± 18.6
	M2	44.5 ± 32.9	47.7 ± 30.9	31.0 ± 14.7	43.8 ± 31.0
	M3	31.8 ± 10.8	38.5 ± 11.4	42.5 ± 30.0	36.5 ± 23,2

*SD* standard deviation, *FPI-6* Foot Posture Index 6, *ARI* Arch Rigidity Index, *CoP* center of pressure, *CoP EA* CoP ellipse area, *M1* measurement point 1 (baseline), *M2* measurement point 2 (end of intervention period), *M3* measurement point 3 (end of wash-out period).

For the ARI, the likelihood-ratio test showed no significant result for the comparison of the model with and without interaction between the group and measurement time point for the trained (χ^2^(2) = 1.10, p = .58) and the untrained leg (χ^2^(2) = 0.33, p = .85), which means that there was no significant difference between the groups for changes over time.

The changes of the results over time for the CoP path length and the ellipse area of the CoP differed significantly between groups for the trained leg (CoP path length: χ^2^(2) = 8.22, p = .02; ellipse area CoP: χ^2^(2) = 6.03, p = .05), but not the untrained leg (CoP path length: χ^2^(2) = 1.86, p = 0.40; ellipse area CoP: χ^2^(2) = 2.57, p = .28). The total linear model with interaction showed a reduction of the CoP path length from M1 to M2 of around 14% in the FE group (β -47.74 (44.00)), though it was not statistically significant (Appendix, Table 1). It only became significant at the reduction in CoP path length from M1 to M3 (around 25%) (β = −  128.16 (45.16)) (t(50) = − 2.84, p = .007). The effect plot of the CoP path length linear regression results is shown in Fig. 3. There were no significant results for the ellipse area of the CoP (Appendix, Table 1).

Descriptive data for the foot parameter are presented in Table 2. The total linear model results with (FPI-6, trained leg CoP path length, and ellipse area) or without interaction (ARI, untrained leg CoP path length, and ellipse area) are listed in the Appendix (Appendix, Table 1).

### Range of motion and Flexibility

The comparison of the linear models for the first metatarsophalangeal joint ROM did not show a significant result for either the trained (χ^2^(2) = 0.54, p = .76) or for the untrained leg (χ^2^(2) = 1.12, p = .57). The linear model without interaction showed a significant change from M1 to M2 and M3, which did not differ significantly between groups (Appendix, Table 2).

For ankle ROM of the trained leg, the likelihood-ratio test indicated that outcome score changes between the measurement points are significantly different between groups (χ^2^(2) = 6.74, p = .03), but not for the untrained leg (χ^2^(2) = 0.81, p = 0.67). For the trained leg, there was an increase of around 15% from M1 to M2 for the FE group (β = 1.32 (0.72)), but the change over time in comparison to the control group was only trending towards significance (t(50) = 1.81, p = 0.08) and only became significant at M3 (β = 1.80 (0.72)) (t(50) = 2.49, p = 0.02). The effect plot of the ankle ROM linear regression results for the trained or first tested leg is shown in Fig. 3.

The comparison of the linear models for the PC flexibility did not show a significant result for either the trained (χ^2^(2) = 0.93, p = .63) or the untrained leg (χ^2^(2) = 1.34, p = .51). Descriptive data for the ROM and flexibility parameter are presented in Table 3. The results of the linear models with (ankle ROM) and without interaction (MTPJ 1, ankle ROM, PC flexibility) are listed in the Appendix (Appendix, Table 2).

**Table 3:** Descriptive data of the range of motion parameters for both groups (foot exercise and control) at all three measurement points.

Variable	Measurement	Foot exercise (mean ± SD)		Control (mean ± SD)	
		Trained	Untrained	First tested	Second tested
MTPJ1 (°)	M1	99 ± 14	94 ± 16	99 ± 16	98 ± 17
	M2	92 ± 14	89 ± 18	95 ± 16	88 ± 16
	M3	90 ± 12	89 ± 14	93 ± 15	90 ± 16
Ankle (Ktw)	M1	12.4 ± 4.8	13.3 ± 5.1	13.1 ± 3.5	12.5 ± 3.2
	M2	14.3 ± 4.6	14.7 ± 4.1	13.2 ± 3.3	12.9 ± 3.5
	M3	13.4 ± 4.9	13.0 ± 5.6	12.5 ± 3.3	12.6 ± 3.5
PC (SaR)	M1	28.5 ± 9.43	28.5 ± 9.3	33.8 ± 11.5	35.4 ± 11.1
	M2	30.4 ± 7.4	29.5 ± 7.5	34.9 ± 9.7	35.8 ± 9.5
	M3	29.8 ± 8.2	29.2 ± 8.1	33.7 ± 10.3	34.9 ± 10.4

*SD* standard deviation, *MTPJ1* metatarsophalangeal joint 1, *KtW* Knee-to-wall test score, *PC* posterior chain, *SaR* Sit-and-reach test score; M1, measurement point 1 (baseline), *M2* measurement point 2 (end of intervention period), *M3* measurement point 3 (end of wash-out period).

### Performance posterior chain

The performance PC outcome changes over time did not differ significantly between groups for the trained (Bunkie Test: χ^2^(2) = 3.30, p = .19; Isometric Posterior Chain Test: χ^2^(2) = 2.78, p = .25; isokinetic measurement 60°/s: (χ^2^(2) = 1.24, p = .54; isokinetic measurement 120°/s: χ^2^(2) = 2.56, p = .28) and the untrained leg (Bunkie Test: χ^2^(2) = 1.45, p = .49; Isometric Posterior Chain Test: χ^2^(2) = 3.46, p = .18; isokinetic measurement 60°/s: χ^2^(2) = 1.65, p = .44; isokinetic measurement 120°/s: χ^2^(2) = 2.56, p = .28). Descriptive data for the PC performance parameters are presented in Table 4. The results of the linear models without interaction are listed in the Appendix (Appendix, Table 3).

**Table 4:** Descriptive data of the strength of the dorsal chain for both groups (foot exercise and control) at all three measurement points.

Variable	Measurement	Foot exercise (mean ± SD)		Control (mean ± SD)	
		Trained	Untrained	First tested	Second tested
Bunkie Test (s)	M1	21.5 ± 13.3	18.9 ± 10.5	15.8 ± 7.6	16.0 ± 7.5
	M2	29.8 ± 11.5	23.7 ± 10.4	17.3 ± 8.2	17.1 ± 8.0
	M3	30.4 ± 10.3	28.7 ± 9.9	22.8 ± 11.9	22.5 ± 11.2
IPC [N]	M1	303.8 ± 77.6	296.1 ± 66.9	275.7 ± 86.5	281.7 ± 80.2
	M2	320.6 ± 78.6	300.4 ± 66.6	271.7 ± 87.4	265.7 ± 81.7
	M3	331.6 ± 80.1	325.9 ± 63.8	296.8 ± 101.2	287.2 ± 98.1
Hamstrings 60°/s (Nm)	M1	103.2 ± 36.0	97.0 ± 31.6	95.1 ± 28.5	89.1 ± 21.8
	M2	105.0 ± 37.6	102.2 ± 34.2	90.1 ± 27.3	86.7 ± 25.7
	M3	102.6 ± 41.6	98.0 ± 35.4	86.6 ± 23.0	88.7 ± 25.0
Hamstrings 120°/s (Nm)	M1	92.2 ± 32.9	87.0 ± 30.9	84.5 ± 23.3	83.3 ± 23.8
	M2	96.8 ± 38.2	93.5 ± 33.9	79.6 ± 24.5	80.7 ± 22.6
	M3	97.0 ± 41.5	93.7 ± 38.2	82.5 ± 25.0	82.3 ± 25.5

*SD* standard deviation, *IPC* Standing 90:20 Isometric Posterior Chain Test, *M1* measurement point 1 (baseline), *M2* measurement point 2 (end of intervention period), *M3* measurement point 3 (end of wash-out period).

## Discussion

The results only partly confirm our hypothesis, that four weeks of FE has local and remote effects in the PC and foot parameters. Foot posture improved in both the trained and the untrained leg after the four-week FE intervention, but the changes were only maintained in the trained leg after the four-week wash-out phase. The CoP path length improved after the FE intervention, but the changes only differed significantly from those of the control group after the wash-out phase (M3). Similarly, FE positively affected ankle ROM, but the changes over time compared to the control group only became significant at M3. In this study, we did not observe any further chronic contralateral or remote effects of FE on the PC concerning ROM and performance.

### Local effects on the foot

We found an improvement in FPI-6 after four weeks of FE intervention in the trained leg, which was maintained after four weeks without FE. Similar to our study, in a clinical population with pes planus, six weeks of short foot exercises improved FPI-6^14^. Sánchez-Rodríguez, et al.^60^ also report an improvement in FPI-6 after participation in a more general exercise program (i.e., foot-, leg-, trunk muscles) for eight weeks. In the study by Matias, et al.^61^, runners also performed eight weeks of training of the foot and ankle muscles^20^, which improved foot biomechanics during running. Although prior studies report similar results, we do not believe that only four weeks of solely training the IFM changes foot anatomy in a generally healthy population without foot deformities. Further, the crossover effect observed for foot posture was not seen in all other parameters. This leads to the assumption that body awareness concerning foot posture improved, and participants intuitively 'corrected' foot posture during standing.

Interestingly, ankle ROM only improved significantly in the trained leg from M1 to M3 (i.e., only trending towards significance at M2), which could be explained by the chosen statistical analysis method. The analysis compares the magnitude of changes between measurement points between groups, which only became significantly different at M3 (see Fig. 3). Nevertheless, the descriptive data already shows an improvement in ROM from M1 to M2 (Table 3).

The ARI did not change after four weeks of FE. Similar to our study, Lynn, et al.^15^ found no significant effect of four weeks of IFM training on medial longitudinal arch mobility measured via navicular drop test. In contrast, a five-week program was reported to be beneficial for clinical populations (i.e., flat foot) for medial longitudinal arch mobility^1^. A longer intervention duration (i.e., eight weeks) might have led to greater medial longitudinal arch mobility changes^3,61,62^. In their review, de Souza, et al.^4^ highlight the differences in medial longitudinal arch mobility measurements and methodological weaknesses in prior studies as potential reasons for differences in outcomes.

The single-leg static balance test results are ambiguous. While no effect of FE was found on the CoP ellipse area, participants of the intervention group could reduce the CoP path length, but these changes only differed significantly between groups between M2 and M3. Notably, the results of FE on balance are inconsistent. Kim and Lee^63^, for example found positive effects of five weeks of short foot exercises on static balance in participants with flat foot but not in healthy participants. Lynn, et al.^15^ reported improvements in dynamic stability for four weeks of IFM training. Mulligan and Cook^3^ report positive effects of short foot exercises on both dynamic and static balance, with greater effects on dynamic balance.

A possible, explanation for the results obtained in our study is that the sample we studied could not further reduce body sway, as some postural sway is necessary for effectivemotor control^64^. Additionally, the improvement in motor control of the IFM during the intervention might have enhanced the somatosensory integration, resulting in more controlled CoP movement within the participant’s natural sway area.

Studies have shown beneficial outcomes for the short foot exercise concerning foot posture^14^, medial longitudinal arch mobility^1,3^, and dynamic stability^3,15^. While IFM muscle activity was measured to be high during the short foot exercise, especially in the abductor hallucis muscle in a study by Jung, et al.^18^, Kim, et al.^65^ showed comparably higher IFM activation during toe spread-out FE. Therefore, our intervention consisted of a total bout of FE to increase the training effect for all IFM^17^. Generally, according to prior studies, clinical populations seem to benefit more from FE than healthy participants^1,14,63^.

### Remote effects along the PC

In contrast to the local effects, little is known about the impact of IFM FE on remote effects. Sulowska, et al.^37^ reported remote improvements in fundamental movement patterns after six weeks, yet only for the group that performed FE including training of the total leg. Sulowska, et al.^38^ also investigated six weeks of FE in runners and found increased knee flexor torque at 90°/s. Yet, it must be mentioned that participants also performed standing FE, e.g., reverse tandem gait^38^. In another study, Sulowska-Daszyk, et al.^36^ reported improvements in functional muscle tests in the leg and lower back muscles (i.e., piriformis, tensor fasciae latae, adductor muscles, and quadratus lumborum) and fundamental movement patterns, measured via functional movement screen after six weeks of FE (again FE included the total leg)^36^. These results contrast our study, yet these studies^36–38^ are hardly comparable to this work: the interventions included standing FE, the intervention duration was longer, the outcome measures differed from our study, and two of the studies were performed on runners^36,38^. Further, participants performed five minutes of foam rolling on the plantar foot sole before the FE^36,38^, which was shown to acutely and chronically influence remote PC ROM^28,29,66–70^. In another study, Goo, et al.^34^ found significantly increased remote gluteus maximus muscle activity after training the abductor hallucis muscle and the gluteus maximus muscle for four weeks, but, similar to our study, there was no remote PC effect after training the abductor hallucis muscle alone.

Although the positive acute and remote PC ROM effects after FR, stretching and manual techniques on the plantar foot sole are well investigated^28,29,31^, there is little evidence for positive chronic effects^69^. In contrast, Konrad, et al.^30^ report no effect of a combination of stretching and foam rolling for seven weeks on ROM and strength parameters of the calf muscles. The remote effects of plantar foot sole treatment on performance are not investigated, yet Gabriel, et al.^71^ report even a negative performance effect of a combined foam rolling, stretching, and manual technique intervention. In contrast, little is known about the remote effects of active interventions, like IFM FE, on the performance and ROM of the PC. While myofascial force transmission effects of neurophysiological changes (i.e. state of arousal, pain/stretch tolerance) are discussed for the remote PC flexibility changes after FR, stretching, and manual technique interventions on the plantar foot sole, the rationale for remote PC effects after FE remains unclear.

### Contralateral effects

In contrast to the positive ipsilateral remote effects of foam rolling on the plantar foot sole on the PC^28,29^, Grabow, et al.^72^ report that acute contralateral ankle and PC ROM were not affected by a foam rolling intervention on the plantar foot sole. There is, to the authors’ best knowledge, little evidence concerning performance changes in the contralateral leg after interventions on the plantar foot sole^73^. Gabriel, et al.^71^ also found no acute performance changes in the PC of the contralateral leg after a combined foam rolling, stretching and manual technique intervention on the plantar foot sole. According to the results of our study, we cannot confirm any chronic contralateral effects resulting from an active intervention targeting the plantar foot structures.

### Foot exercise intervention

In prior literature, the duration of FE interventions varies between four to eight weeks^1,3,12,14–16,19,38^, which were comparably more extended training periods than in this study. The training frequency in studies varies between seven^12,14,38^ and four days per week^16^, similar to our study. Various studies report that some FE trainings per week were performed under supervision^12,14,38^. Other FE interventions were completely unsupervised^3,15^. According to a review by de Souza, et al.^4^, the training volume in most studies ranges between five and ten seconds of isometric contraction and three to five series for each exercise, which is similar to our research. They also conclude that it is still being determined whether this is the best training protocol and if exercise progression from sitting to standing is the best type of progression^4^. Jung, et al.^18^ report higher activation of the abductor hallucis muscle when IFM FE are performed standing compared to sitting, which might explain the higher effects on foot parameters reported in prior studies. Some studies report that the difficulty of the exercises progressed after one^12^ or two weeks^14,15,38^, or when subjects felt that they could perform the exercise properly without great exertion^3,12^. The progression mainly includes changing the exercise position from sitting to standing and/or including different training tools^3,12,15,18,38^. In our study, we chose the progression of the exercises by adding resistance with an elastic band (TheraBand, Ludwig Artzt GmbH, GE) to guarantee that FE mainly targeted IFM and not the PC muscles.

In general, IFM FE are time-consuming and contain coordinatively demanding elements, which might lower exercise adherence and motivation in participants. Therefore, other interventions, such as walking in minimalist shoes, should be considered as alternative or add-on intervention, as it seems to be similarly effective in increasing IFM size^74^ and showed similar positive local effects on foot posture and balance for an intervention duration of four weeks^33^.

### Limitations

Compared to prior studies, the duration of the intervention was short, which might have led to a lower magnitude of changes. To guarantee that the IFM are mainly targeted during the FE, FE progression was not implemented by changing position to standing but by adding resistance with an elastic band (TheraBand, Ludwig Artzt GmbH, GE)^12^, which might have lowered the effect of FE. Further, we would like to point out that the distribution of sexes was not equal between groups in this study.

## Conclusion

Our study shows that four weeks of FE might be beneficial for recreationally active young adults as foot posture, balance, and ankle ROM improved. Interestingly, foot posture also improved in the contralateral, non-trained leg. In contrast to prior studies, we did not observe any remote flexibility or performance effects along the PC after active FE targeting the plantar foot. The rationale for remote effects after active FE interventions on the plantar foot sole, reported by prior studies, should be further discussed.

## Supplementary Information

Supplementary Information.

## Data Availability

All data are available upon request from the corresponding author.

## References

[1] Kim, E.-K. & Kim, J. S. The effects of short foot exercises and arch support insoles on improvement in the medial longitudinal arch and dynamic balance of flexible flatfoot patients. *J. Phys. Ther. Sci.* **28**, 3136–3139 (2016). 27942135 10.1589/jpts.28.3136 PMC5140815

[2] Kelly, L. A., Cresswell, A. G., Racinais, S., Whiteley, R. & Lichtwark, G. Intrinsic foot muscles have the capacity to control deformation of the longitudinal arch. *J. R. Soc. Interface* **11**, 20131188 (2014). 24478287 10.1098/rsif.2013.1188 PMC3928948

[3] Mulligan, E. P. & Cook, P. G. Effect of plantar intrinsic muscle training on medial longitudinal arch morphology and dynamic function. *Man. Ther.* **18**, 425–430 (2013). 23632367 10.1016/j.math.2013.02.007

[4] de Souza, T. M. M., de Oliveira Coutinho, V. G., Tessutti, V. D., de Oliveira, N. R. C. & Yi, L. C. Effects of intrinsic foot muscle strengthening on the medial longitudinal arch mobility and function: A systematic review. *J. Bodyw. Mov. Ther.* **36**, 89–99 (2023). 37949605 10.1016/j.jbmt.2023.05.010

[5] Curtis, R., Willems, C., Paoletti, P. & D’Août, K. Daily activity in minimal footwear increases foot strength. *Sci. Rep.* **11**, 1–10 (2021). 34545114 10.1038/s41598-021-98070-0 PMC8452613

[6] Davis, I. S. *et al.* Stepping back to minimal footwear: Applications across the lifespan. *Exerc. Sport Sci. Rev.* **49**, 228–243 (2021). 34091498 10.1249/JES.0000000000000263

[7] Hollander, K., Heidt, C., Van der Zwaard, B. C., Braumann, K.-M. & Zech, A. Long-term effects of habitual barefoot running and walking: A systematic review. *Med. Sci. Sports Exerc.* **49**, 752–762 (2017). 27801744 10.1249/MSS.0000000000001141

[8] Hannigan, J. & Pollard, C. D. Comparing walking biomechanics of older females in maximal, minimal, and traditional shoes. *Gait Posture* **83**, 245–249 (2021). 33197860 10.1016/j.gaitpost.2020.10.030

[9] Huber, G., Jaitner, T. & Schmidt, M. Acute effects of minimalist shoes on biomechanical gait parameters in comparison to walking barefoot and in cushioned shoes: A randomised crossover study. *Footwear Sci.* **14**, 123–130 (2022).

[10] Holowka, N. B., Wallace, I. J. & Lieberman, D. E. Foot strength and stiffness are related to footwear use in a comparison of minimally-vs conventionally-shod populations. *Sci. Rep.* **8**, 1–12 (2018). 29487321 10.1038/s41598-018-21916-7 PMC5829167

[11] Tourillon, R., Gojanovic, B. & Fourchet, F. How to evaluate and improve foot strength in athletes: An update. *Front. Sports Active Liv.* **1**, 46 (2019). 10.3389/fspor.2019.00046 PMC7739583 33344969

[12] Fraser, J. J. & Hertel, J. Effects of a 4-week intrinsic foot muscle exercise program on motor function: A preliminary randomized control trial. *J. Sport Rehabil.* **28**, 339–349 (2019). 29364026 10.1123/jsr.2017-0150

[13] Ridge, S. T. *et al.**Walking in Minimalist Shoes is Effective for Strengthening Foot Muscles* (2018). 10.1249/MSS.0000000000001751 30113521

[14] Unver, B., Erdem, E. U. & Akbas, E. Effects of short-foot exercises on foot posture, pain, disability, and plantar pressure in pes planus. *J. Sport Rehabil.* **29**, 436–440 (2019). 30860412 10.1123/jsr.2018-0363

[15] Lynn, S. K., Padilla, R. A. & Tsang, K. K. Differences in static-and dynamic-balance task performance after 4 weeks of intrinsic-foot-muscle training: The short-foot exercise versus the towel-curl exercise. *J. Sport Rehabil.* **21**, 327–333 (2012). 22715143 10.1123/jsr.21.4.327

[16] Goldmann, J.-P., Sanno, M., Willwacher, S., Heinrich, K. & Brüggemann, G.-P. The potential of toe flexor muscles to enhance performance. *J. Sports Sci.* **31**, 424–433 (2013). 23106289 10.1080/02640414.2012.736627

[17] Gooding, T. M., Feger, M. A., Hart, J. M. & Hertel, J. Intrinsic foot muscle activation during specific exercises: A T2 time magnetic resonance imaging study. *J. Athl. Train.* **51**, 644–650 (2016). 27690528 10.4085/1062-6050-51.10.07 PMC5094843

[18] Jung, D.-Y. *et al.* A comparison in the muscle activity of the abductor hallucis and the medial longitudinal arch angle during toe curl and short foot exercises. *Phys. Ther. Sport* **12**, 30–35 (2011). 21256447 10.1016/j.ptsp.2010.08.001

[19] Taddei, U. T. *et al.* Effects of a therapeutic foot exercise program on injury incidence, foot functionality and biomechanics in long-distance runners: Feasibility study for a randomized controlled trial. *Phys. Ther. Sport* **34**, 216–226 (2018). 30388670 10.1016/j.ptsp.2018.10.015

[20] Matias, A. B., Taddei, U. T., Duarte, M. & Sacco, I. C. Protocol for evaluating the effects of a therapeutic foot exercise program on injury incidence, foot functionality and biomechanics in long-distance runners: A randomized controlled trial. *BMC Musculoskelet. Disord.* **17**, 1–11 (2016). 27075480 10.1186/s12891-016-1016-9 PMC4831173

[21] Wei, Z., Zeng, Z., Liu, M. & Wang, L. Effect of intrinsic foot muscles training on foot function and dynamic postural balance: A systematic review and meta-analysis. *PLoS ONE* **17**, e0266525 (2022). 35442981 10.1371/journal.pone.0266525 PMC9020712

[22] McKeon, P. O., Hertel, J., Bramble, D. & Davis, I. The foot core system: A new paradigm for understanding intrinsic foot muscle function. *Br. J. Sports Med.* **49**, 290–290 (2015). 24659509 10.1136/bjsports-2013-092690

[23] Krause, F., Wilke, J., Vogt, L. & Banzer, W. Intermuscular force transmission along myofascial chains: A systematic review. *J. Anat.* **228**, 910–918 (2016). 27001027 10.1111/joa.12464 PMC5341578

[24] Gabriel, A., Paternoster, F. K., Konrad, A., Horstmann, T. & Pohl, T. Comparison between the original-and a standardized version of a physical assessment test for the dorsal chain: A cohort-based cross sectional study. *J. Sports Sci. Med.* **21**, 182–190 (2022). 35719223 10.52082/jssm.2022.182 PMC9157515

[25] Gabriel, A. K. *et al.* Testing the posterior chain: Diagnostic Accuracy of the Bunkie test versus the isokinetic hamstrings/quadriceps measurement in patients with self-reported knee pain and healthy controls. *Clinical Medicine* **13**, 1–14. 10.3390/jcm13041011 (2024). 10.3390/jcm13041011 PMC10889369 38398324

[26] Myers, T. W. *Anatomy Trains e-book: Myofascial Meridians for Manual and Movement Therapists* (Elsevier Health Sciences, 2013).

[27] Wilke, J., Krause, F., Vogt, L. & Banzer, W. What is evidence-based about myofascial chains: A systematic review. *Arch. Phys. Med. Rehabil.* **97**, 454–461 (2016). 26281953 10.1016/j.apmr.2015.07.023

[28] Burk, C., Perry, J., Lis, S., Dischiavi, S. & Bleakley, C. Can myofascial interventions have a remote effect on ROM? A systematic review and meta-analysis. *J. Sport Rehabil.* **29**, 650–656 (2019). 31629335 10.1123/jsr.2019-0074

[29] Dhiman, N. R. *et al.* Myofascial release versus other soft tissue release techniques along superficial back line structures for improving flexibility in asymptomatic adults: A systematic review with meta-analysis. *J. Bodyw. Mov. Ther.* **28**, 450–457 (2021). 34776177 10.1016/j.jbmt.2021.06.026

[30] Konrad, A. *et al.* Remote effects of a 7-week combined stretching and foam rolling training intervention of the plantar foot sole on the function and structure of the triceps surae. *Eur. J. Appl. Physiol.* **123**, 1645–1653 (2023). 36973555 10.1007/s00421-023-05185-5 PMC10363033

[31] Gala, M., Kulkarni, P. & Kumar, A. Comparison of immediate effect of plantar fascia release by roller massager and transverse friction massage on hamstring flexibility in desk job workers. *Int. J. Physiother. Res.* **9**, 3954–3959 (2021).

[32] Snow, N., Basset, F. & Byrne, J. An acute bout of barefoot running alters lower-limb muscle activation for minimalist shoe users. *Int. J. Sports Med.* **37**, 382–387 (2016). 26837932 10.1055/s-0035-1565140

[33] Gabriel, A. *et al.* A four-week minimalist shoe walking intervention influences foot posture and balance in young adults–a randomized controlled trial. *PLoS ONE* **19**, e0304640 (2024). 38900749 10.1371/journal.pone.0304640 PMC11189255

[34] Goo, Y.-M., Kim, T.-H. & Lim, J.-Y. The effects of gluteus maximus and abductor hallucis strengthening exercises for four weeks on navicular drop and lower extremity muscle activity during gait with flatfoot. *J. Phys. Ther. Sci.* **28**, 911–915 (2016). 27134383 10.1589/jpts.28.911 PMC4842464

[35] Azevedo, A. P. D. S., Mezêncio, B., Amadio, A. C. & Serrao, J. C. 16 weeks of progressive barefoot running training changes impact force and muscle activation in habitual shod runners. *PLoS ONE* **11**, e0167234 (2016). 27907069 10.1371/journal.pone.0167234 PMC5132300

[36] Sulowska-Daszyk, I., Mika, A. & Oleksy, Ł. Impact of short foot muscle exercises on quality of movement and flexibility in amateur runners. *Int. J. Environ. Res. Public Health* **17**, 6534 (2020). 32911733 10.3390/ijerph17186534 PMC7558208

[37] Sulowska, I., Oleksy, Ł, Mika, A., Bylina, D. & Sołtan, J. The influence of plantar short foot muscle exercises on foot posture and fundamental movement patterns in long-distance runners, a non-randomized, non-blinded clinical trial. *PLoS ONE* **11**, e0157917 (2016). 27336689 10.1371/journal.pone.0157917 PMC4918976

[38] Sulowska, I., Mika, A., Oleksy, Ł & Stolarczyk, A. The influence of plantar short foot muscle exercises on the lower extremity muscle strength and power in proximal segments of the kinematic chain in long-distance runners. *BioMed Res. Int.* **2019**, 1–11 (2019). 10.1155/2019/6947273 PMC6334361 30719446

[39] Tudor-Locke, C. *et al.* How many steps/day are enough? For adults. *Int. J. Behav. Nutr. Phys. Act.* **8**, 1–17 (2011).21194492

[40] Lin, H. *Effect size converter*, <https://www.escal.site/> (2023).

[41] RANDOM.ORG. *Random Integer Generator*. www.random.org (2022).

[42] Van Melick, N., Meddeler, B. M., Hoogeboom, T. J., Nijhuis-van der Sanden, M. W. & Van Cingel, R. E. How to determine leg dominance: The agreement between self-reported and observed performance in healthy adults. *PLoS ONE* **12**, e01898076 (2017). 10.1371/journal.pone.0189876 PMC5747428 29287067

[43] Redmond, A., Burns, J., Crosbie, J., Ouvrier, R. & Peat, J. An initial appraisal of the validity of a criterion based, observational clinical rating system for foot posture. *J. Orthop. Sports Phys. Ther.* **31**, 160 (2001).

[44] Keenan, A.-M., Redmond, A. C., Horton, M., Conaghan, P. G. & Tennant, A. The Foot Posture Index: Rasch analysis of a novel, foot-specific outcome measure. *Arch. Phys. Med. Rehabil.* **88**, 88–93 (2007). 17207681 10.1016/j.apmr.2006.10.005

[45] Vulcano, E., Tracey, J. A. III. & Myerson, M. S. Accurate measurement of first metatarsophalangeal range of motion in patients with hallux rigidus. *Foot Ankle Int.* **37**, 537–541 (2016). 26660863 10.1177/1071100715621508

[46] Allan, J. J. *et al.* First metatarsophalangeal joint range of motion is associated with lower limb kinematics in individuals with first metatarsophalangeal joint osteoarthritis. *J. Foot Ankle Res.* **13**, 1–8 (2020). 32513212 10.1186/s13047-020-00404-0 PMC7278053

[47] Powden, C. J., Hoch, J. M. & Hoch, M. C. Reliability and minimal detectable change of the weight-bearing lunge test: A systematic review. *Man. Ther.* **20**, 524–532 (2015). 25704110 10.1016/j.math.2015.01.004

[48] López-Miñarro, P. A., de Baranda Andújar, P. S. & RodrÑGuez-GarcÑa, P. L. A comparison of the sit-and-reach test and the back-saver sit-and-reach test in university students. *J. Sports Sci. Med.* **8**, 116 (2009). 24150564 PMC3737781

[49] De Witt, B. & Venter, R. The ‘Bunkie’ test: Assessing functional strength to restore function through fascia manipulation. *J. Bodyw. Mov. Ther.* **13**, 81–88 (2009). 19118796 10.1016/j.jbmt.2008.04.035

[50] Brumitt, J. The Bunkie test: Descriptive data for a novel test of core muscular endurance. *Rehabil. Res. Pract.* **2015**, 1–9 (2015). 10.1155/2015/780127 PMC4339703 25852955

[51] Ronai, P. The bunkie test. *Strength & Conditioning Journal* **37**, 89–92 (2015).

[52] Van Pletzen, D. & Venter, R. E. The relationship between the bunkie-test and physical performance in rugby union players. *Int. J. Sports Sci. Coach.* **7**, 543–553 (2012).

[53] Matinlauri, A. *et al.* A comparison of the isometric force fatigue-recovery profile in two posterior chain lower limb tests following simulated soccer competition. *PLoS ONE* **14**, e0206561 (2019). 31050674 10.1371/journal.pone.0206561 PMC6499418

[54] Emrani, A. *et al.* Isokinetic strength and functional status in knee osteoarthritis. *J. Phys. Ther. Sci.* **18**, 107–114 (2006).

[55] Grygorowicz, M., Kubacki, J., Pilis, W., Gieremek, K. & Rzepka, R. Selected isokinetic tests in knee injury prevention. *Biol. Sport* **27**, 47–51 (2010).

[56] Jeon, K., Seo, B.-D. & Lee, S.-H. Comparative study on isokinetic capacity of knee and ankle joints by functional injury. *Journal of physical therapy science* **28**, 250–256 (2016). 26957768 10.1589/jpts.28.250 PMC4756014

[57] Rosene, J. M., Fogarty, T. D. & Mahaffey, B. L. Isokinetic hamstrings: Quadriceps ratios in intercollegiate athletes. *J. Athl. Train.* **36**, 378 (2001). 12937479 PMC155432

[58] *R: A Language and Environment for Statistical Computing* (R Foundation for Statistical Computing, 2018). https://www.R-project.org/.

[59] Kuznetsova, A., Brockhoff, P. B. & Christensen, R. H. lmerTest package: Tests in linear mixed effects models. *J. Stat. Softw.* **82**, 1–26 (2017).

[60] Sánchez-Rodríguez, R. *et al.* Modification of pronated foot posture after a program of therapeutic exercises. *Int. J. Environ. Res. Public Health* **17**, 8406 (2020). 33202893 10.3390/ijerph17228406 PMC7697388

[61] Matias, A. B. *et al.* Effects of foot-core training on foot-ankle kinematics and running kinetics in runners: Secondary outcomes from a randomized controlled trial. *Front. Bioeng. Biotechnol.* **10**, 890428 (2022). 35497357 10.3389/fbioe.2022.890428 PMC9046605

[62] Chung, K. A., Lee, E. & Lee, S. The effect of intrinsic foot muscle training on medial longitudinal arch and ankle stability in patients with chronic ankle sprain accompanied by foot pronation. *Phys. Ther. Rehabil. Sci.* **5**, 78–83 (2016).

[63] Kim, J. S. & Lee, M. Y. The effect of short foot exercise using visual feedback on the balance and accuracy of knee joint movement in subjects with flexible flatfoot. *Medicine* **99**, e19260 (2020). 32221061 10.1097/MD.0000000000019260 PMC7220527

[64] Riccio, G. E. Information in movement variability about qualitative dynamics of posture and orientation. *Variability and Motor Control* (1993).

[65] Kim, M.-H., Kwon, O.-Y., Kim, S.-H. & Jung, D.-Y. Comparison of muscle activities of abductor hallucis and adductor hallucis between the short foot and toe-spread-out exercises in subjects with mild hallux valgus. *J. Back Musculoskelet. Rehabil.* **26**, 163–168 (2013). 23640317 10.3233/BMR-2012-00363

[66] Grieve, R. *et al.* The immediate effect of bilateral self myofascial release on the plantar surface of the feet on hamstring and lumbar spine flexibility: A pilot randomised controlled trial. *J. Bodyw. Mov. Ther.* **19**, 544–552 (2015). 26118527 10.1016/j.jbmt.2014.12.004

[67] Patel, D. G., Vyas, N. J. & Sheth, M. S. Immediate effect of application of bilateral self myo-fascial release on the plantar surface of the foot on hamstring and lumbar spine flexibility: A quasi experimental study. *Foot* **3**, 94–99 (2016).

[68] Kwangsun, D., Jaeeun, K. & Jongeun, Y. Acute effect of self-myofascial release using a foam roller on the plantar fascia on hamstring and lumbar spine superficial back line flexibility. *Phys. Ther. Rehabil. Sci.* **7**, 35–40 (2018).

[69] Shetty, K. & Dsouza, M. R. Effectiveness of plantar fascia mobilization and passive stretching on hamstring muscle flexibility. *Int. J. Health Sci. Res.* **8**, 134–137 (2018).

[70] Joshi, D. G., Balthillaya, G. & Prabhu, A. Effect of remote myofascial release on hamstring flexibility in asymptomatic individuals–A randomized clinical trial. *J. Bodywork Mov. Ther.* **22**, 832–837 (2018). 10.1016/j.jbmt.2018.01.008 30100320

[71] Gabriel, A. *et al.* Myofascial treatment techniques on the plantar surface influence functional performance in the dorsal kinetic chain. *J. Sports Sci. Med.* **21**, 13–22 (2021). 10.52082/jssm.2022.13 PMC8851122 35250329

[72] Grabow, L., Young, J. D., Byrne, J. M., Granacher, U. & Behm, D. G. Unilateral rolling of the foot did not affect non-local range of motion or balance. *J. Sports Sci. Med.* **16**, 209 (2017). 28630574 PMC5465983

[73] Konrad, A., Nakamura, M., Warneke, K., Donti, O. & Gabriel, A. The contralateral effects of foam rolling on range of motion and muscle performance. *Eur. J. Appl. Physiol.* **123**, 1167–1178 (2023). 36694004 10.1007/s00421-023-05142-2 PMC10191906

[74] Ridge, S. *et al.* A comparison of foot strengthening versus minimal footwear use on intrinsic muscle size and strength. *Foot Ankle Orthop.* **3**, 2473011418S2473000406 (2018).

