# Supplementary material for: Local and non-local effects (on the posterior chain) of four weeks of foot exercises: a randomized controlled trial
Source: Sci Rep. 2024 Sep 23;14:22000. doi: 10.1038/s41598-024-71585-y (PMC11422494; doi:10.1038/s41598-024-71585-y)
Supplement: Supplementary file 1 — Supplementary Information. [file 41598_2024_71585_MOESM1_ESM.docx]

**APPENDIX**

Table 1. Linear regression model results of the foot parameters for the trained and the untrained leg

|  | **Trained leg** | | | **Untrained leg** | |  |
| --- | --- | --- | --- | --- | --- | --- |
| *Predictors* | *Estimates* | *CI* | *p* | *Estimates* | *CI* | *p* |
| **Foot Posture Index 6** | | | | | | |
| Intercept | 24.07 | 22.46 – 25.68 |  | 24.60 | 22.96 – 26.24 |  |
| Intervention | 1.07 | -1.21 – 3.35 | .35 | -0.07 | -2.39 – 2.25 | .96 |
| M2 | 0.46 | -0.81 – 1.74 | .47 | -0.75 | -1.99 – 0.49 | .23 |
| M3 | -1.36 | -2.67 – -0.05 | .04* | -1.31 | -2.59 – -0.04 | .04* |
| intervention * M2 | -4.77 | -6.57 – -2.97 | <.001* | -2.10 | -3.86 – -0.34 | .02* |
| intervention * M3 | -2.16 | -3.98 – -0.33 | .02* | -0.68 | -2.46 – 1.11 | .45 |
| **Arch Rigidity Index** | | | | | | |
| Intercept | 0.92 | 0.91 – 0.94 |  | 0.92 | 0.90 – 0.94 |  |
| Intervention | -0.01 | -0.03 – 0.01 | 0.44 | 0.01 | -0.01 – 0.03 | .47 |
| M2 | 0.01 | -0.01 – 0.03 | 0.45 | 0.01 | -0.01 – 0.03 | .24 |
| M3 | 0.00 | -0.02 – 0.03 | 0.77 | -0.01 | -0.02 – 0.01 | .56 |
| **Center of Pressure Ellipse Area** | | | | | | |
| Intercept | 33.96 | 22.16 – 45.77 |  | 34.62 | 22.41 – 46.83 |  |
| Intervention | 5.61 | -10.81 – 22.02 | .50 | 8.40 | -7.03 – 23.83 | .28 |
| M2 | -2.96 | -17.40 – 11.47 | .68 | 7.18 | -2.55 – 16.90 | .15 |
| M3 | 8.45 | -6.30 – 23.21 | .26 | -1.48 | -11.33 – 8.37 | .77 |
| intervention * M2 | 7.89 | -12.29 – 28.08 | .44 |  |  |  |
| intervention * M3 | -16.63 | -37.27 – 4.00 | .11 |  |  |  |
| **Center of Pressure Distance** | | | | | | |
| Intercept | 363.19 | 287.59 – 438.80 |  | 351.23 | 267.36 – 435.09 |  |
| Intervention | 86.38 | -19.60 – 192.35 | .11 | 55.05 | -58.98 – 169.07 | .34 |
| M2 | -14.62 | -77.42 – 48.18 | .64 | -2.45 | -43.50 – 38.60 | .91 |
| M3 | 8.37 | -56.11 – 72.85 | .80 | -33.47 | -75.09 – 8.15 | .11 |
| intervention * M2 | -47.74 | -135.41 – 39.93 | .28 |  |  |  |
| intervention * M3 | -128.16 | -218.17 – -38.16 | .006* |  |  |  |
| Note: CI, confidence interval; p, p-value; M2, measurement two (after four weeks intervention); M3, measurement three (after four weeks wash-out); * indicates a statistically significant result (p ≤ .05) | | | | | | |

Table 2. Linear regression model results of the range of motion parameters for the trained and the untrained leg

|  | **Trained leg** | | | **Untrained leg** | |  |
| --- | --- | --- | --- | --- | --- | --- |
| *Predictors* | *Estimates* | *CI* | *p* | *Estimates* | *CI* | *p* |
| **Metatarsophalangeal Joint One** | | | | | | |
| Intercept | 100.13 | 93.15 – 107.10 |  | 97.10 | 89.20 – 104.99 |  |
| Intervention | -1.38 | -10.55 – 7.79 | .77 | -2.00 | -12.38 – 8.39 | .70 |
| M2 | -5.14 | -9.96 – -0.31 | .04* | -6.72 | -12.14 – -1.30 | .02* |
| M3 | -7.17 | -11.99 – -2.35 | .004* | -5.75 | -11.16 – -0.33 | .04* |
| **Ankle Joint** | | | | | | |
| Intercept | 13.07 | 10.92 – 15.21 |  | 12.56 | 10.32 – 14.79 |  |
| Intervention | -0.63 | -3.67 – 2.40 | .68 | 0.68 | -2.45 – 3.81 | .67 |
| M2 | 0.02 | -0.99 – 1.02 | .98 | 0.34 | -0.23 – 0.90 | .24 |
| M3 | -0.64 | -1.67 – 0.39 | .22 | -0.19 | -0.75 – 0.38 | .51 |
| intervention * M2 | 1.31 | -0.13 – 2.75 | .08 |  |  |  |
| intervention * M3 | 1.80 | 0.36 – 3.24 | .02* |  |  |  |
| **Total Posterior Chain** | | | | | | |
| Intercept | 33.48 | 28.60 – 38.37 |  | 35.05 | 30.17 – 39.92 |  |
| Intervention | -4.64 | -11.47 – 2.20 | .18 | -6.13 | -12.95 – 0.69 | .08 |
| M2 | 2.28 | 0.91 – 3.65 | .001* | 1.51 | 0.23 – 2.80 | .02* |
| M3 | 1.57 | 0.18 – 2.96 | .03* | 1.10 | -0.20 – 2.40 | .10 |
| Note: CI, confidence interval; p, p-value; M2, measurement two (after four weeks intervention); M3, measurement three (after four weeks wash-out); * indicates a statistically significant result (p ≤ .05) | | | | | | |

Table 3. Linear regression model results of the performance parameters for the trained and the untrained leg

|  | **Trained leg** | | | **Untrained leg** | |  |
| --- | --- | --- | --- | --- | --- | --- |
| *Predictors* | *Estimates* | *CI* | *p* | *Estimates* | *CI* | *p* |
| **Bunkie Test** | | | | | | |
| Intercept | 14.43 | 9.43 – 19.44 |  | 14.88 | 10.31 – 19.45 |  |
| Intervention | 8.47 | 2.07 – 14.86 | .01* | 5.10 | -0.77 – 10.98 | .09 |
| M2 | 4.43 | 0.44 – 8.42 | .03* | 2.43 | -1.12 – 5.97 | .18 |
| M3 | 7.66 | 3.67 – 11.65 | <0.001* | 8.18 | 4.63 – 11.72 | <0.001* |
| **90:20 Isometric Posterior Chain Test** | | | | | | |
| Intercept | 271.78 | 229.21 – 314.36 |  | 275.41 | 237.57 – 313.25 |  |
| Intervention | 35.93 | -23.69 – 95.54 | .23 | 26.96 | -25.64 – 79.57 | .31 |
| M2 | 2.32 | -8.83 – 13.46 | .68 | -8.80 | -22.04 – 4.45 | .19 |
| M3 | 18.66 | 7.20 – 30.12 | .002* | 12.28 | -1.36 – 25.91 | .08 |
| **Isokinetic Measurement Hamstrings at 60°/s** | | | | | | |
| Intercept | 93.61 | 77.31 – 109.92 |  | 88.23 | 73.79 – 102.68 |  |
| Intervention | 11.30 | -11.17 – 33.77 | .32 | 10.36 | -9.46 – 30.18 | .30 |
| M2 | -2.56 | -7.11 – 2.00 | .27 | 0.72 | -4.12 – 5.56 | .77 |
| M3 | -4.61 | -9.16 – -0.06 | .05* | -0.65 | -5.49 – 4.19 | .79 |
| **Isokinetic Measurement Hamstrings at 120°/s** | | | | | | |
| Intercept | 82.64 | 67.05 – 98.23 |  | 81.31 | 66.66 – 95.96 |  |
| Intervention | 12.11 | -9.33 – 33.56 | .26 | 9.02 | -11.08 – 29.12 | .37 |
| M2 | -0.93 | -5.63 – 3.78 | 0.70 | 1.36 | -3.56 – 6.29 | .58 |
| M3 | 0.76 | -3.94 – 5.47 | 0.75 | 2.09 | -2.83 – 7.01 | .40 |
| Note: CI, confidence interval; p, p-value; M2, measurement two (after four weeks intervention); M3, measurement three (after four weeks wash-out); * indicates a statistically significant result (p ≤ .05) | | | | | | |
